# Ames Assay Transferred from the Microtiter Plate to the Planar Assay Format

**DOI:** 10.3390/jox15030067

**Published:** 2025-05-07

**Authors:** Katharina Schmidtmann, Johanna Lemme, Gertrud E. Morlock

**Affiliations:** Chair of Food Science, Institute of Nutritional Science, Justus Liebig University Giessen, Heinrich-Buff-Ring 26-32, 35392 Giessen, Germany; katharina.schmidtmann@ernaehrung.uni-giessen.de (K.S.); lemme.johanna@web.de (J.L.)

**Keywords:** high-performance thin-layer chromatography (HPTLC), cancer, rapid assay, 4-nitrochinolin-*N*-oxide

## Abstract

The International Agency for Research on Cancer has studied and classified 1045 potential substances. It is therefore important to develop rapid screening methods to identify the mutagenicity of compounds and, further on, the intensity and number of individual mutagenic substances in complex sample mixtures. The current in vitro Ames assay in the microtiter plate format (MPF) uses a pH-sensitive detection as endpoint, however, acidic substances in complex mixtures may interfere the mutagenicity result. Hence, it was transferred to the planar assay format to be more selective for complex mixture testing. The co-culture of *Salmonella* Typhimurium strains TA98 and TA100 with an optical density of 0.4 at 600 nm was applied on a high-performance thin-layer chromatography silica gel 60 chromatogram and on-surface incubated for 5 h, which period was limited due to zone diffusion. Various positive controls were tested, and 4-nitrochinolin-*N*-oxide with a limit of detection of 100 ng was established as a positive control. However, due to the shorter incubation time, no mutagenic compounds were detectable or differentiable in the tested perfumes, herbal teas, margarines, and hand creams. This does not mean that the samples are mutagen-free, but it suggests that further improvements to the bioassay are urgently needed to increase the sensitivity and selectivity of the response. Compared to conventional sum value assays, a planar Ames assay performed on the separated and adsorbed sample components advances toxicology research because mutagenic compounds are separated from interfering molecules due to the integrated separation. It thus would provide a more selective detection of mutagens in complex mixtures and allow testing of large sample volumes or concentrated samples without matrix interference.

## 1. Introduction

Chronic noncommunicable diseases, including cancer, are responsible for 74% of deaths globally. Cancer is widespread, and the second most common cause of death after cardiovascular diseases [1]. In the year 2022, the global incidence of cancer was approximately 19.9 million cases across both sexes, with an associated mortality rate of around 9.7 million deaths [2,3,4]. The International Agency for Research on Cancer has studied and classified 1045 cancer-potential substances. The carcinogenic effect could not be classified for 500 agents, whereas 128 agents were carcinogenic, 95 agents were probably carcinogenic, and 323 agents were possibly carcinogenic to humans [5]. Testing more chemicals for their mutagenic potential is important to reduce the high case numbers.

In the early 1970s, Ames et al. [6,7,8,9,10] developed a sensitive and simple in vitro bacterial test for detecting potential mutagens or carcinogens. The Ames test is described in the OECD 471 guideline as the bacterial reverse mutation test [11]. The assay utilized distinct strains of *S*. Typhimurium, containing specific histidine mutations [7,8]. The OECD recommends the use of five reliable strains of *Salmonella* Typhimurium (TA1535, TA1537, TA97, TA97a, TA98, and TA100) because of their reproducible response between different laboratories [11,12]. Williams et al. [13,14] determined that the strains TA98 and TA100 detected 93% of the tested mutagens. The TA100 strain has a hisG46 mutation, which is responsible for histidine biosynthesis [8], whereas the TA98 strain has a hisD3052 mutation preventing the encoding of the histidinol dehydrogenase in the hisD gene [15]. Both auxotropic strains need histidine in the medium for growth. Mutagens in a sample can mutate the bacteria to grow in a histidine-free medium, which is measured. Further development of the Ames test led to the in vitro Ames microtiter plate format (MPF) assay using 96-well plates instead of agar plates [16]. The detection of mutagenic compounds in the Ames MPF was based on the associated decrease in the pH caused by the active metabolism (producing acidic compounds) of the revertant prototrophic *Salmonella* strains [16,17,18,19,20], leading to a chromatic shift of an added pH indicator compound, such as bromocresol purple from purple to yellow at pK_1_ = 5.2 [21].

The Ames MPF assay is recommended and limited to studying only single compounds and not complex samples, as acidic components, false-positives or false-negatives may falsify the sum value and thus mislead the decision. Using another assay, only individual (single) ingredients of fragrances, not the whole perfume, were investigated via the BlueScreen HC microtiter plate in vitro assay using p53-competent human TK6 lymphoblastoid cells and the Gaussia luciferase reporter system for luminescence detection of genotoxicants and compared with the in vivo micronucleus test and 3D skin-based micronucleus assay [22]. There is great demand for screening techniques able to study complex sample mixtures directly, including the intensity and number of individual mutagenic ingredients or xenogens. The screening should be fast and cost-efficient. The hyphenation of high-performance thin-layer chromatography (HPTLC) with planar bioassays offers such potential. Its distinct advantages in comparison to microtiter plate in vitro assays are the faster workflow and the separation from interfering compounds (e.g., with false-negative and false-positive responses) and other compounds with opposing effects (e.g., cytotoxins and antagonists). Overall, it directly enables the detection of individual active compounds in a complex sample as well as their straightforward characterization [23,24]. For example, using the planar SOS-UmuC-FLD bioassay, genotoxic compounds were directly detected in a highly selective and sensitive manner and characterized in complex samples such as food contact materials [25,26,27], healthy oils [28,29], cosmetics [30,31], and perfumes [32,33]. The SOS-Umu-C-FLD bioassay detects genotoxicity through the activation of the SOS response, while the more specific Ames bioassay detects mutagenicity through point mutations and frameshifts. Thus, both bioassays complement each other by covering a broad spectrum of mutagenic effects using specific bacterial strains.

In this study, the Ames MPF assay with two *S*. Typhimurium strains, TA100 and TA98, was transferred to the planar assay format. Therefore, different parameters were tested on an HPTLC silica gel 60 plate, and various mutagenic positive controls [34] were investigated. The planar Ames assay would enable a direct separation of mutagens from interfering compounds, allowing for a more selective and faster detection of individual mutagenic compounds in complex sample matrices compared to the limited conventional Ames assays, including the microtiter plate format.

## 2. Materials and Methods

### 2.1. Chemicals and Materials

cis-Diamineplatinum (II)-dichloride (CP), 4-nitroquinoline-N-oxide (4NQO, >98%), phenyl glycidyl ether (PGE, >99%), and triglycidyl isocyanurate (TIC, >98%) were obtained from Tokyo Chemical Industry (Tokyo, Japan). Methylmethanesulfonate (MMS, 98.7%) was delivered by Santa Cruz Biotechnology (Dallas, TX, USA). N4-Aminocytidine (N4ACT, 95%) was purchased from Angene Chemical (London, UK). N-Nitroso-N-ethylurea (ENU) and glycerol (>99%) were acquired from Sigma Aldrich (St. Louis, MO, USA). Ampicillin sodium salt (>99%) and dimethylsulfoxide (>99.8%) were obtained from Carl Roth (Karlsruhe, Germany). Cyclohexane (99.8%) was from Thermo Fisher Scientific (Waltham, MA, USA). Ethyl acetate (>99.8%) was obtained from Th. Geyer (Renningen, Germany). Methanol was delivered by VWR International (Darmstadt, Germany). Potassium hydroxide (KOH) and HPTLC silica gel 60 plates (20 cm × 10 cm) were supplied by Merck (Darmstadt, Germany). The two *Salmonella* Typhimurium strains, lyophilized and stabilized on a disc format (lyoph. STDisc), i.e., TA98 and TA100, were purchased from Trinova Biochem (Giessen, Germany). The MOLTOX FT Exposure Media, i.e., Exposure Medium, the Oxoid Nutrient Broth No. 2 (growth medium), and the MOLTOX FT Reversion Indicator Medium (indicator medium) were from MOLTOX (Boone, NC, USA). Bi-distilled water was generated using a Heraeus Destamat B-18E (Thermo Fisher Scientific). The following samples were bought in local supermarkets or drugstores: three perfumes (Black, Prada Milan, Italy; Wanted by Night, Azzaro Parfums, Paris, France; and Rush, Gucci, Florence, Italy), two herbal teas (Organic 9-herbs tea, Lord Nelson, Düsseldorf, Germany and 9-herbs tea, King’s Crown, Hamburg, Germany), two margarines (Sanella and Rama, both Upfield, Hamburg, Germany), and two skin creams (Dr. Grandel Elements of Nature—Regeneration, Dr. Grandel, Augsburg, Germany, and Cum Natura Propolis Hand Cream, Cum Natura, Bad Grönenbach, Germany).

### 2.2. Cultivation of the Two Bacterial Strains Using Cryostocks

The cryopreserved cell discs of the two strains of *Salmonella* Typhimurium, TA100 and TA98, were incubated separately in growth medium (Oxoid Nutrient Broth No. 2, 35 mL) at 37 °C and 120 rpm (Edmund Bühler, Tübingen, Germany) for 16 h in an orbital shaker incubator (Cultura M, 70700R, Almedica, Galmiz, Switzerland). Cryostocks were generated by centrifuging (Centrifuge 5702, Eppendorf, Hamburg, Germany) an aliquot of the cell suspension (20 mL, 3000× *g*, 10 min), followed by the removal of the supernatant. The resulting cell pellet was resuspended with ice-cooled growth medium (18 mL) and glycerol (2 mL), subsequently filled into cryovials (1 mL aliquots), and stored at −80 °C. On the day before the analysis, the cryostock supernatant was discarded. As an overnight culture, each strain (25 µL) was incubated separately with the growth medium (35 mL) containing ampicillin solution (37 µL, 100 mg/mL) at 37 °C and 120 rpm for 14–16 h.

### 2.3. Plate Pretreatment

The HPTLC silica gel 60 plates were pre-washed with methanol–water 4:1 (*V*/*V*). Exemplarily, four pieces of the same plate batch were measured to have a mean pH of 6.4 (SenTix SUR, Xylem Analytics, Weilheim, Germany). Thus, each plate was adjusted to pH 7.9 with 3% KOH solution (piezoelectrical spraying of 2.5 mL, blue nozzle, level 3, Derivatizer, CAMAG, Muttenz, Switzerland) and dried at 120 °C for 15 min (TLC Plate Heater III, CAMAG).

### 2.4. Preparation and Testing of the Positive Control Standards as Band Pattern

*cis*-Diamineplatinum (II)-dichloride (CP, 10 mg/mL) and *N*4-aminocytidine (N4ACT, 1 mg/mL) were each dissolved in 30 µL dimethyl sulfoxide, followed by the addition of 970 µL methanol and dilution with methanol to obtain solutions of 5 mg/µL for CP and 0.5 mg/mL for N4ACT. A methanolic solution of 4-nitroquinoline-*N*-oxide (4NQO, 1 mg/mL) was diluted 1:10 with methanol (100 µg/mL) and methyl methanesulfonate (MMS, 400 mg/mL), *N*-nitroso-*N*-ethylurea (ENU, 100 mg/mL), and phenyl glycidyl ether (PGE, 60 mg/mL); each were diluted to obtain solutions of 1 mg/mL.

The tested positive control standards (CP 1–500 µg/band, MMS 1–10,000 µg/band, PGE 1–3000 µg/band, N4ACT 0.4–20 µg/band, ENU 1–10,000 µg/band, and 4NQO 0.3–2 µg/band) were applied as an 8-mm band pattern with the Automatic TLC Sampler 4 (CAMAG, operated by winCATS 1.4.7 software with FreeMode option, with the following settings: syringe, 10 µL; dosage speed, 150 nL/s; predosage volume, 200 nL; filling speed, 15 µL/s; filling vacuum time, 4 s; and rinsing time, 4 s).

### 2.5. Preparation and Separation of the Four Different Sample Categories

Samples were prepared as follows (stored in the refrigerator, if needed). The solutions were applied as 8 mm bands (ATS4 parameters as mentioned) onto the HPTLC silica gel 60 plate, dried (hairdryer, 1 min), developed with the indicated solvent system up to 70 mm, and dried again (hairdryer, 5 min). The plate was detected at white light illumination (visible light, Vis) and FLD 366 nm (Visualizer 2, CAMAG). All HPTLC instrumentation was operated by visionCATS software version 3.2 (CAMAG).

- The perfumes were transferred to vials without any sample preparation, applied (0.8, 1, 10, and 20 µL/band each), and developed with 7 mL of cyclohexane–ethyl acetate 19:1 (*V*/*V*) [33].
- The margarines and skin creams (100 mg each) were dissolved in 1.5 mL of isopropanol–pentane 1:1 (*V/V*), ultrasonificated (Sonorex Digiplus, Bandelin, Berlin, Germany) for 10 min, and centrifuged (17,000× *g*, 10 min). Each upper phase was transferred to a vial, where 5 µL was applied. Development was performed using 5 mL of pentane–diethyl ether 8:3 (*V/V*) after chamber saturation with 20 mL of the solvent system [31].
- The tea samples (500 mg each) were extracted with 5 mL of methanol, ultrasonicated (75 °C, 30 min), and centrifuged (17,000× *g*, 15 min). Each supernatant was filtered (sterile) into vials, where 50 µL was applied and developed using 7 mL of dichloromethane–methanol–ammonia 85:15:1 (*V*/*V*/*V*).

### 2.6. Planar Ames Bioassay Detection

Four bands of the positive control 4NQO (0.1, 0.3, 0.6, and 1 µg/band) were applied above the solvent front at 80 mm. As a negative control chromatogram, all steps were performed as usual, but the inoculation with *Salmonella* cells was skipped. Each overnight culture of auxotropic *S*. Typhimurium strains TA98 and TA100 (which require histidine for growing but cannot produce it) was diluted with the growth medium (containing histidine) to an optical density at 600 nm (OD_600_) of 0.4 (Spectronic CamSpec, West Yorkshire, UK). The cell suspension (1.25 mL of each strain) was pipetted in a 50 mL centrifugation tube (Sarstedt, Nümbrecht, Germany) and centrifuged at 3000× *g* for 5 min; then, the supernatant was discarded. The exposure medium (2.5 mL, histidine-free) was used for cell resuspension and incubated at 37 °C for 40 min, followed by centrifugation at 3000× *g* and discarding the supernatant. The indicator medium (2.5 mL, detection of acidic compounds as endpoint) was used for cell resuspension, and the entire cell suspension was piezoelectrically sprayed (red nozzle, level 3, Derivatizer) on the HPTLC silica gel 60 plate. The seeded plate was placed in a polypropylene box (KIS 26.5 cm × 16 cm × 19 cm, ABM, Wolframs-Eschenbach, Germany; lined with filter paper pre-moistened with 40 mL distilled water) and incubated at 37 °C for 5 h. The plate was dried (5 min, hairdryer) and detected at Vis and FLD 366 nm (Visualizer 2, CAMAG). Mutagenic compounds were visible as yellow zones on a purple background at Vis and slightly blue-fluorescent zones at FLD 366 nm. All assay results were repeated at least twice.

## 3. Results and Discussion

### 3.1. Development of the Planar Ames Assay

**Figure 1:**
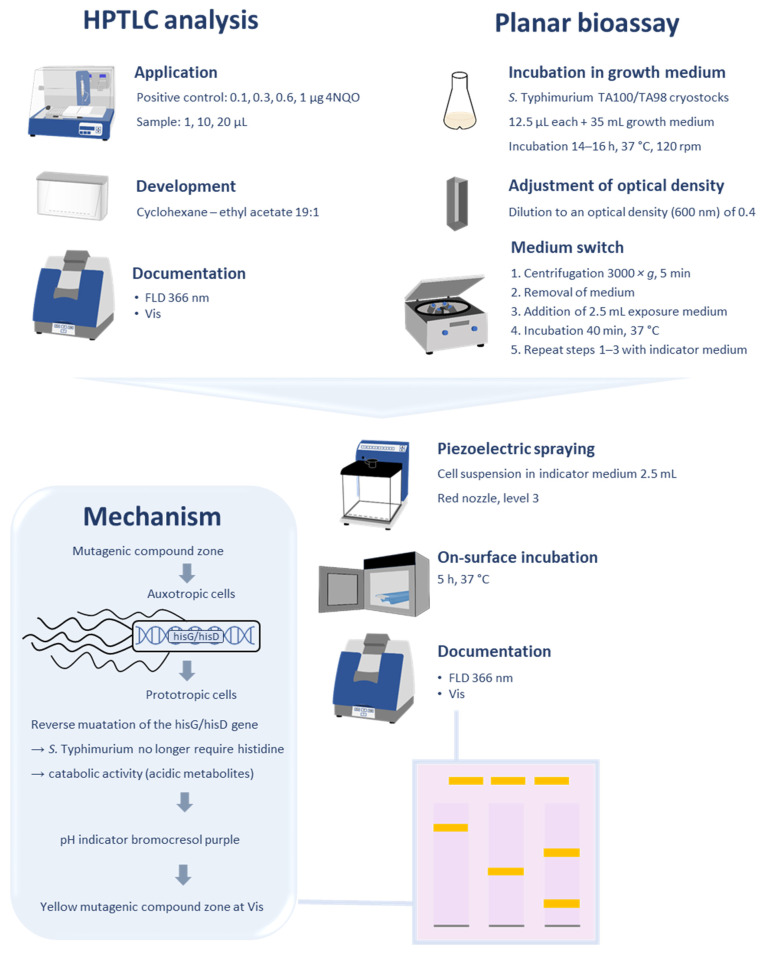
Workflow and parameters of the final planar Ames bioassay method (HPTLC–UV/Vis/FLD–Ames bioassay) performed analogous to the Ames MPF assay. The illustrated mutagenicity mechanism provides a yellow endpoint due to acidic metabolites formed by the catabolic activity of reverted or mutated cells through mutagenic compounds.

The Ames MPF assay [16,17] was the basis for developing the planar Ames bioassay on the HPTLC silica gel 60 plate. Frequently used in mutagenic and genotoxic assays [34], 4NQO was selected as the positive control. Each auxotropic *S*. Typhimurium strain (TA98 and TA100) in the histidine-free exposure medium was individually applied onto an HPTLC plate, followed by incubation for 40 min, subsequent spraying of the pH indicator medium bromocresol purple onto the plate, and incubation for 5 h. In this period, mutagens mutate cells to become prototrophic and produce acidic metabolism products. Unfortunately, no color change in the pH indicator and thus, no visible difference between the 4NQO zone and background was observed. Different factors of influence were tested, such as the pH value of the HPTLC plate, incubation time, OD_600_, and affinity of the two strains towards 4NQO (data not depicted). However, the on-surface incubation in the exposure medium did not work. Instead, the incubation in the exposure medium was then performed in the centrifugation tube during the medium switch, which was not an extra step. After centrifugation, the cell pellet was resuspended with the indicator medium before piezoelectric spraying onto the chromatogram, followed by on-surface incubation for 40 min (Figure 1). Again, the plate pH value, incubation time, cell density OD_600_, and affinity of the two strains towards 4NQO were studied, as well as a negative control.

#### 3.1.1. Adjustment of the Plate pH

The catabolic activity of the reverted or mutated *Salmonella*, which were prototrophic and able to produce histidine, thus exhibiting viability associated with a pH decrease [15,17], led to a color change in the indicator bromocresol purple from purple to yellow at pK_1_ = 5.2 in the Ames MPF assay [21]. In the opposite case, the non-reverted *Salmonella* did not survive in the histidine-deficient medium; thus, the indicator remained purple. However, a slightly acidic pH value of the HPTLC plate (pH 6.4) impaired the detection of the pH change; thus, it had to be adjusted to an alkaline value.

**Figure 2:**
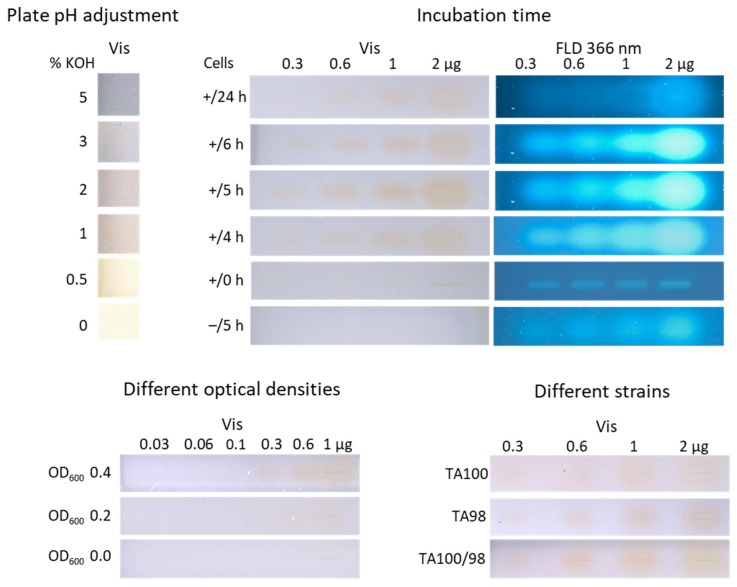
Results of the factors of influence: pH adjustment of the HPTLC silica gel 60 plate treated without (0%) and with five different KOH concentrations (0.5–5%); different incubation times (0–24 h) of the positive control 4NQO (0.3–2 µg/band) with *S.* Typhimurium TA100 (+) versus without (–); different OD_600_ (0.0–0.4) of strain TA100 culture incubated with 4NQO (0.06–1 µg/band) for 5 h; and individual TA100 and TA98 strains versus combined strains TA98/TA100 (1:1) incubated with 4NQO (0.3–2.0 µg/band) for 5 h, all detected after the planar Ames bioassay at Vis and FLD 366 nm.

Five differently concentrated KOH solutions (0.5–5%) were tested (Figure 2). Both the non-treated plate (pH 6.4) and the plate treated with 0.5% KOH solution showed a yellow color, which hindered the detection of the pH change and, thus, the color change from purple to yellow by mutagenic compound zones. Treatment with 1% and 2% KOH solutions turned the plate background into a soft purple. Following the treatment with a 3% KOH solution, an optimal purple coloration was achieved while maintaining a moderate plate pH of 7.9. The more alkaline milieu should not influence cell metabolism as *S*. Typhimurium cells grow in a wide pH range up to pH 9 [35]. Spraying the 5% KOH solution resulted in a plate pH of 9.3, which was too alkaline for cell growth.

#### 3.1.2. Study of the Incubation Time

Another important parameter was the incubation time. The original Ames MPF workflow had an incubation time of 48 h [17]. However, such a long incubation time would cause zone diffusion, which counteracts sensitive detection. To improve sensitivity for a 48 h incubation, the zone diffusion on normal phase plates can be reduced by zone fixation [36], which, however, is focus of another study. Different shorter incubation times (0–24 h) were investigated instead for the strain TA100 and different amounts of 4NQO (0.3–2.0 µg/band). No incubation time (0 h) revealed no yellow 4NQO zone at Vis but a slight blue fluorescence at FLD 366 nm (Figure 2). By increasing the incubation time, differences in the color intensity were observed, with a maximum intensity for 5 h. The 5 h incubation time was found to be acceptable for 4NQO and the strain TA100.

#### 3.1.3. Negative Control as Proof of the Selectivity or Specificity of the Response

Further, it must be excluded that the different ingredients of the *Salmonella* medium, or buffer salts, binder, and impurities within the adsorbent layer, cause a spectral shift of the pH indicator in the presence of 4NQO, which could also generate the yellow color. Thus, as a negative control, a plate was incubated for 5 h without inoculation with *Salmonella*, and no color change to yellow was detected in the bioautogram at Vis (Figure 2). However, the more sensitive detection bioautogram at FLD 366 nm revealed slightly blue fluorescent zones despite the absence of cells. This observation was studied in detail with further mutagenic positive controls (3.2).

#### 3.1.4. Adjustment of the Optical Density of the Cell Culture

As a third parameter, different ODs of the cell culture (OD_600_ 0.0–0.4) were tested. The bioautograms for OD_600_ values of 0.2 and 0.4 showed a rising yellow intensity with increasing 4NQO amounts (0.06–1 µg/band; Figure 2). The higher OD_600_ of 0.4 was chosen as it led to a stronger color intensity for the 4NQO amounts of 0.3–1 µg/band.

#### 3.1.5. Detection Limit of Individual *Versus* Combined Strains Towards 4NQO

Using the optimized parameters of the planar Ames assay, the individual affinity of the strains TA98 and TA100 was tested versus their combined affinity (TA98/TA100, 1:1) towards 4NQO (0.3–2 µg/band). Almost no difference was observed in the bioautograms at Vis (Figure 2). The combination and utilization of both strains enabled an efficient and fast workflow. The visual limit of detection (LOD) of 4NQO was determined to be 100 ng/band for both strains. This LOD is 10 times less sensitive for 4NQO than the Ames MPF assay, with the lowest effective concentration (LEC) of 1 ng and 10 ng for the strains TA 100 and TA 98, respectively [34]. This might be explained by the shorter 5 h on-surface incubation, which reduces *Salmonella* reversion opportunities, compromising sensitivity, in comparison to the 48 h incubation in the Ames MPF assay.

A direct comparison between the two different assay technologies is challenging due to fundamental differences, i.e., the Ames MPF operates in the liquid phase (reports concentrations), while the planar assay works on the adsorbent surface (reports amounts). Thus, a 10% hypothetical stock solution was used to compare the Ames MPF and planar Ames assay systems. In the Ames MPF, 10 µL of the stock solution (1 mg) is applied per well and diluted in a total volume of 2850 µL, as per standard protocols. This results in a 285-fold dilution of the stock solution. This significantly reduces the effective exposure of bacterial cells to the test compound, which may limit the detection of weakly mutagenic substances. In contrast, in the planar Ames assay, 100 µL of the 10% stock solution (10 mg) can be applied onto a defined surface area (e.g., 8 mm band). Without further dilution, this ensures that bacterial cells are exposed to a 285-fold higher actual amount compared to the Ames MPF system. Although 2.5 mL of bacterial suspension is sprayed onto the plate, the test compound remains undiluted, as it is adsorbed. A greater potential for detecting weakly mutagenic compounds is thus suggested for the planar Ames assay, although the current short 5 h incubation counteracts this.

### 3.2. Screening of Further Mutagenic Reference Substances

Slightly blue fluorescent signals of 4NQO were already observed in the bioassay milieu in the absence of *Salmonella* cells and, thus, without metabolic activity (Figure 2); however, the signals substantially increased in the presence of cells and showed a clear signal difference and, thus, mutagenicity. Contamination with any microorganisms, which could produce acidic metabolites and cause a pH shift, was excluded by heating, i.e., sterilizing (120 °C, 20 min), the plate before applying the positive controls.

**Figure 3:**
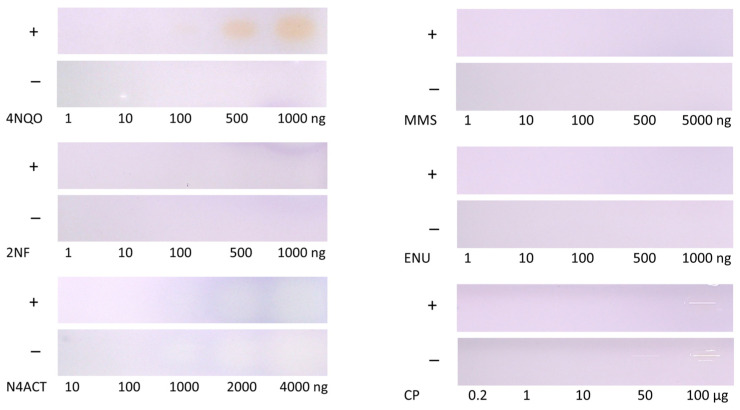
Comparison of five different mutagenic positive controls detected at Vis after the planar Ames bioassay with (+) versus without (−) the combined *S.* Typhimurium strains TA98/TA100: ENU (1–1000 ng/band), MMS (1–5000 ng/band), CP (0.2–100 µg/band), *N*4ACT (10–4000 ng/band), 2NF (1–1000 ng/band), and 4NQO (1–1000 ng/band) detectable at 100 ng/zone. The planar bioassay sensitivity was worse due to the short 5 h incubation time compared to the Ames MPF assay (48 h).

Different amounts of standards were tested as positive controls [34], with and without *Salmonella* strains TA98 and TA100. For the positive controls, CP, ENU, MMS, and 2NF, no clear differentiation with and without cell treatment was possible (Figure 3). A possible reason could be that the incubation time was too short (5 h compared to the usual 48 h). *Salmonella* may require a longer incubation period to form revertants. The literature reported 4NQO as the most sensitive positive control for both *Salmonella* strains [34]. This might explain why 4NQO yielded a positive response in comparison to the other mutagenic references. The positive control N4ACT revealed a yellow-blue color in the absence of both *Salmonella* strains and only a slight increase when present. As the plate was pre-washed, impurities on the plate were reduced, and this should have no impact.

### 3.3. Application to Four Different Sample Categories Pointing to False Positives/Negatives

**Figure 4:**
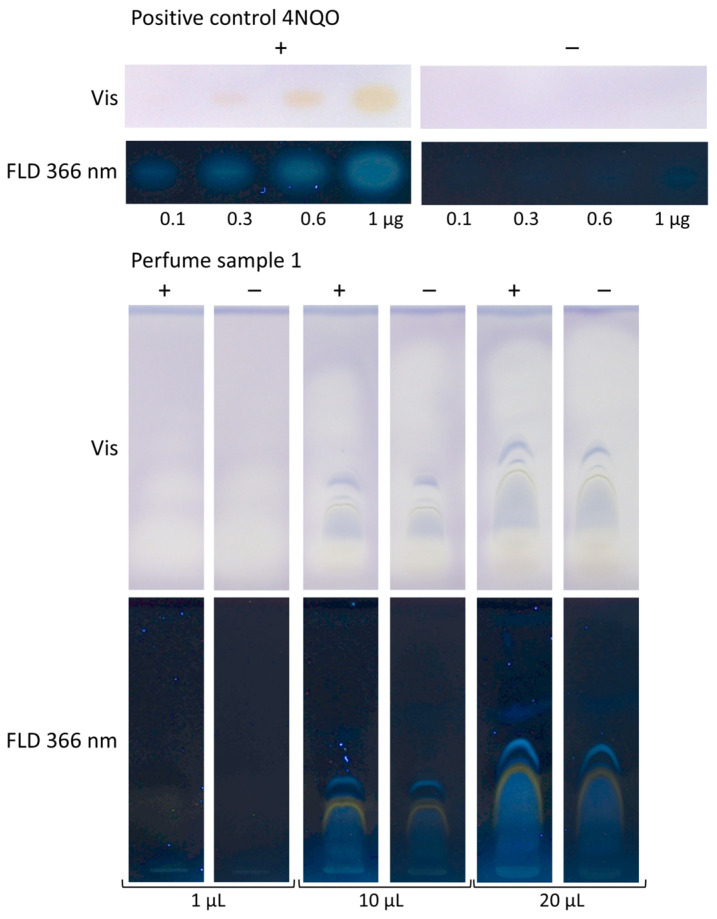
Sample volume selection: different volumes of a perfume (1–20 µL) were developed on the HPTLC silica gel 60 plate with cyclohexane–ethyl acetate 19:1, subjected to the bioassay with *S.* Typhimurium (+) versus without (−), and detected at Vis and FLD 366 nm. In the HPTLC–Ames bioassay–FLD bioautogram (+), comparatively stronger blue fluorescent zones at FLD indicated mutagenicity, in contrast to the negative control chromatogram (−). In the respective Vis bioautogram (+), detection was less sensitive and masked by interference with blue-colored matrix zones. Proper bioassay performance was proven by the positive control 4NQO (0.1–1 µg/band), detected as an increase in yellow-colored or blue fluorescent zones with increasing volumes.

The planar Ames assay enabled direct detection of individual mutagenic compounds in complex samples and a separation from matrix interferences. This is key in contrast to the Ames MPF assay, which provides only a sum value and is prone to false negatives and false positives. Different sample volumes of a perfume sample (1, 10, and 20 µL/band) were analyzed. On the same chromatogram, 4NQO (0.1–1 µg/band) was applied as a positive control above the solvent front. After performing the bioassay, the increasing volumes of the positive control 4NQO showed increasingly yellow-colored and blue fluorescent zones, which proved proper assay performance (Figure 4). In the HPTLC–Ames bioassay–FLD bioautogram (+), mutagenicity was indicated because comparatively stronger blue fluorescent sample zones were detected in contrast to the negative control chromatogram (−). Mutagenicity was evident for all three sample volumes. The absorbance detection was not as sensitive as the fluorescence detection, and it was masked by interference with blue-colored matrix zones, which made the detection of any difference in the yellow zone coloration between the HPTLC–Ames bioassay–Vis bioautogram (+) and the negative control chromatogram (−) obsolete.

**Figure 5:**
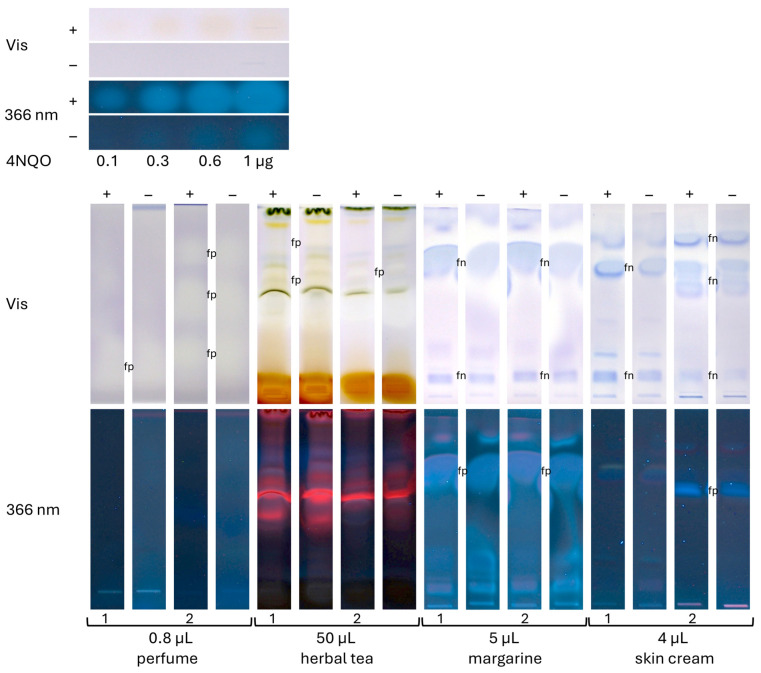
Analysis of two samples each of the four different categories (volumes as indicated) on an HPTLC silica gel 60 plate developed with cyclohexane–ethyl acetate 19:1 (perfumes), dichloromethane–methanol–ammonia 85:15:1 (herbal teas), and pentane–diethyl ether 8:3 (margarines and skin creams). Proper bioassay performance was proven by the positive control 4NQO (0.1–1 µg/band), detected as an increase in yellow zones with increasing volumes; however, for the eight samples, no difference was observed between the HPTLC–Ames bioassay–Vis bioautograms (+) and the negative control chromatogram (−). These planar bioutograms clearly demonstrate the problems of the Ames MPF assay sum value result for the analysis of such complex mixtures with no differentiation of false positives (fp; white/yellow-colored or blue-fluorescent zones) and false negatives (fn; blue-colored).

Two samples each of the four different categories, i.e., perfumes, teas, margarines, and cosmetics, were analyzed using a respective sample volume (selected as exemplarily shown in Figure 4) and mobile phase (as mentioned in Section 2.5). The positive control 4NQO worked properly (Figure 5), showing yellow zones in the HPTLC–Ames bioassay–Vis bioautogram (+) and blue fluorescent zones in the HPTLC–Ames bioassay–FLD bioautogram (+) in contrast to the negative control chromatogram (−). For all eight samples, yellow or slightly yellow zones were detected in the HPTLC–Ames bioassay–Vis and blue or red fluorescent zones in the HPTLC–Ames bioassay–FLD bioautogram (+), but no difference was observed from the negative control chromatogram (−). The yellow sample zones in the negative control chromatogram (−) were explained by acidic compounds, i.e., matrix moieties decreasing the pH. Unfavorably, this was analogous to the detection of mutagenicity indicated by an acidic pH due to the active metabolism of reverted cells. This revealed that the current low selectivity of the Ames MPF assay, which was transferred to the planar format, easily caused false-positive responses for the analysis of mixtures. A potential approach to mitigate false-positive responses of the Ames MPF assay is the removal of interfering acidic matrix components through a targeted sample clean-up, e.g., using liquid–liquid extraction or solid-phase extraction. However, such steps inherently risk sample loss, including mutagenic compounds, potentially compromising assay sensitivity. Alternatively, fluorescence- or luminescence-based detection may improve selectivity.

A comparison between the Ames MPF bioassay and the developed planar Ames bioassay, both indicating mutagenicity, as well as the planar SOS-Umu-C bioassay indicating genotoxicity, was performed (Table 1). It is estimated that the developed planar Ames bioassay is currently due to the short 5 h on-surface incubation about 10 times less sensitive for 4NQO compared with the in vitro Ames MPF bioassay. It is also less sensitive than the planar SOS-Umu-C bioassay used for perfumes [33] and cosmetics [31]. Although the Ames bioassay is well-established for assessing mutagenicity, its reliance on sample dilution to mitigate matrix effects when analyzing complex mixtures can lead to false negatives, as potentially mutagenic compounds may be diluted below their detection limit. In addition, there is no differentiation of mutagens from false positives and false negatives for the analysis of complex mixtures.

## 4. Conclusions

The in vitro Ames MPF bioassay in the microtiter plate format was successfully transferred to the planar bioassay format. It became evident that the currently widespread and frequently used in vitro Ames MPF assay, which provides only a sum value result, would mislead decisions made for samples containing acidic or colored matrix compounds. Depending on the sample, matrix effects can be different and cause different false-positive/-negative effects using the in vitro Ames MPF assay. It is essential to fundamentally reconsider and rethink the Ames MPF assay for the analysis of multi-constituent substances, mixtures, or complex samples, as our study revealed drawbacks and limitations due to the pH indicator endpoint lacking in selectivity. Integrated separation is the key to the analysis of complex mixtures. The proper performance and functionality of the planar HPTLC–UV/Vis/FLD–Ames bioassay was proven via the positive control 4NQO. The incubation time was limited to 5 h due to zone diffusion, which explained its 10-fold worse sensitivity for 4NQO compared to the Ames MPF assay (48 h). Further improvements in sensitivity and selectivity of the planar Ames assay are essential to realize its full potential. To increase the sensitivity, the incubation time could be extended when combined with zone fixation to prevent zone diffusion. To increase the selectivity, the current susceptibility to false positives could be avoided by the implementation of alternative detections, such as fluorescence- or luminescence-based systems instead of the current pH indicator medium. It is urgent to improve the planar Ames bioassay because it offers the advantage of a more detailed analysis of complex samples due to the integrated sample separation and, optionally, direct characterization of mutagenic compound zones by online elution to high-resolution mass spectrometry.

## Figures and Tables

**Table 1:** The drawbacks of the Ames MPF bioassay for the analysis of complex mixtures recognized during transfer to the planar Ames bioassay; for comparison, the planar SOS-Umu-C bioassay is also considered.

Bioassay	Advantages of Complex Mixture Analysis	Disadvantages of Complex Mixture Analysis
Ames MPF (mutagenicity)	None	Not usable for complex mixtures Long incubation time (48 h) Limited to single substances Non-selective for acidic components Prone to matrix effects Dilution can lead to false negatives
Planar Ames (mutagenicity)	Separation from matrix components Selective detection of mutagens	About 10-fold lower sensitivity for 4NQO Partially co-eluting acidic components
Planar SOS-Umu-C (genotoxicity)	Separation from matrix components High sensitivity and selectivity by fluorescence detection Fast analysis (3 h)	Requires devices for fluorescence detection

## Data Availability

The raw data supporting the conclusions of this article will be made available by the authors upon request.

## References

[1] World Health Organization (2023). Noncommunicable Diseases. www.who.int/news-room/fact-sheets/detail/noncommunicable-diseases.

[2] Cancer Today Absolute Numbers, Incidence, Both Sexes, in 2022. https://gco.iarc.who.int/today/en/dataviz/pie?mode=population&group_populations=0.

[3] Bosetti C., Bertuccio P., Malvezzi M., Levi F., Chatenoud L., Negri E., La Vecchia C. (2013). Cancer mortality in Europe, 2005–2009, and an overview of trends since 1980. Ann. Oncol..

[4] Ferlay J., Colombet M., Soerjomataram I., Dyba T., Randi G., Bettio M., Gavin A., Visser O., Bray F. (2018). Cancer incidence and mortality patterns in Europe: Estimates for 40 countries and 25 major cancers in 2018. Eur. J. Cancer.

[5] International Agency for Research on Cancer (IARC) (2023). Agents Classified by the IARC Monographs, Volumes 1–135. https://monographs.iarc.who.int/agents-classified-by-the-iarc/.

[6] Ames B.N., Joyce M., Edith Y. (1975). Methods for Detecting Carcinogens and Mutagens with the *Salmonella*/Mammalian-Microsome Mutagenicity Test. Mutat. Res..

[7] Ames B.N., Durston W.E., Yamasaki E., Lee F.D. (1973). Carcinogens are Mutagens: A Simple Test System Combining Liver Homogenates for Activation and Bacteria for Detection. Proc. Nat. Acad. Sci. USA.

[8] Ames B.N., Charles Y. (1971). The Detection of Chemical Mutagens with Enteric Bacteria. Chemical Mutagens.

[9] Houk V.S., Claxton L.D. (1986). Screening complex hazardous wastes for mutagenic activity using a modified version of the TLC/Salmonella assay. Mutat. Res..

[10] Bjørseth A., Eidså G., Gether J., Landmark L., Møller M. (1982). Detection of mutagens in complex samples by the Salmonella assay applied directly on thin-layer chromatography plates. Science.

[11] OECD (2020). Test No. 471: Bacterial Reverse Mutation Test.

[12] Mortelmans K., Zeiger E. (2000). The Ames Salmonella/microsome mutagenicity assay. Mutat. Res..

[13] Williams R.V., DeMarini D.M., Stankowski L.F., Escobar P.A., Zeiger E., Howe J., Elespuru R., Cross K.P. (2019). Are all bacterial strains required by OECD mutagenicity test guideline TG471 needed?. Mutat. Res. Genet. Toxicol. Environ. Mutagen..

[14] Cross K.P., DeMarini D.M. (2023). Analysis of chemical structures and mutations detected by Salmonella TA98 and TA100. Mutat. Res..

[15] Maron D.M., Ames B.N. (1983). Revised methods for *Salmonella* mutagenicity test. Mutat. Res..

[16] Flückiger-Isler S., Baumeister M., Braun K., Gervais V., Hasler-Nguyen N., Reimann R., van Gompel J., Wunderlich H.-G., Engelhardt G. (2004). Assessment of the performance of the Ames II assay: A collaborative study with 19 coded compounds. Mutat. Res..

[17] Flückiger-Isler S., Kamber M. (2012). Direct comparison of the Ames microplate format (MPF) test in liquid medium with the standard Ames pre-incubation assay on agar plates by use of equivocal to weakly positive test compounds. Mutat. Res..

[18] Flückiger-Isler S., Kamber M. (2006). The Ames MPF 98/100 Assay: Novel Mutagenicity Testing in Liquid Microplate Format Using S. typhimurium TA98 and TA100, (Poster Presentation).

[19] Flückiger-Isler S., Kamber M. (2009). The Ames MPF™ Penta I Assay: Mutagenicity Testing in Liquid Microplate Format Using OECD Guideline 471 Compliant Strains, (Poster Presentation).

[20] Spiliotopoulos D., Koelbert C., Audebert M., Barisch I., Bellet D., Constans M., Czich A., Finot F., Gervais V., Khoury L. (2024). Assessment of the performance of the Ames MPF™ assay: A multicenter collaborative study with six coded chemicals. Mutat. Res. Genet. Toxicol. Environ. Mutagen..

[21] Yao W., Byrne R.H. (2001). Spectrophotometric determination of freshwater pH using bromocresol purple and phenol red. Environ. Sci. Technol..

[22] Thakkar Y., Joshi K., Hickey C., Wahler J., Wall B., Etter S., Smith B., Griem P., Tate M., Jones F. (2022). The BlueScreen HC assay to predict the genotoxic potential of fragrance materials. Mutagenesis.

[23] Morlock G.E. (2021). High-performance thin-layer chromatography combined with effect-directed assays and high-resolution mass spectrometry as an emerging hyphenated technology: A tutorial review. Anal. Chim. Acta.

[24] Morlock G.E. (2022). Planar chromatographic super-hyphenations for rapid dereplication. Phytochem. Rev..

[25] Varalda G.M., Zellmer S., Tietz T. (2024). Risk assessment of food contact materials. EFSA J..

[26] Mayrhofer E., Prielinger L., Sharp V., Rainer B., Kirchnawy C., Rung C., Gruner A., Juric M., Springer A. (2023). Safety Assessment of Recycled Plastics from Post-Consumer Waste with a Combination of a Miniaturized Ames Test and Chromatographic Analysis. Recycling.

[27] Rainer B., Mayrhofer E., Redl M., Dolak I., Mislivececk D., Czerny T., Kirchnawy C., Marin-Kuan M., Schilter B., Tacker M. (2019). Mutagenicity assessment of food contact material migrates with the Ames MPF assay. Food Addit. Contam. Part A Chem. Anal. Control Expo. Risk Assess..

[28] Dung C.-H., Wu S.-C., Yen G.-C. (2006). Genotoxicity and oxidative stress of the mutagenic compounds formed in fumes of heated soybean oil, sunflower oil and lard. Toxicol. In Vitro.

[29] Morlock G.E., Meyer D. (2023). Designed genotoxicity profiling detects genotoxic compounds in staple food such as healthy oils. Food Chem..

[30] Di Sotto A., Maffei F., Hrelia P., Di Giacomo S., Pagano E., Borrelli F., Mazzanti G. (2014). Genotoxicity assessment of some cosmetic and food additives. Regul. Toxicol. Pharmacol..

[31] Morlock G.E., Zoller L. (2025). Fast unmasking toxicity of safe personal care products. J. Chromatogr. A.

[32] Al-Saleh I., Al-Rajudi T., Al-Qudaihi G., Manogaran P. (2017). Evaluating the potential genotoxicity of phthalates esters (PAEs) in perfumes using in vitro assays. Environ. Sci. Pollut. Res. Int..

[33] Morlock G.E., Heil J. (2025). Fast unmasking hazards of safe perfumes. J. Chromatogr. A.

[34] Rainer B., Pinter E., Prielinger L., Coppola C., Marin-Kuan M., Schilter B., Apprich S., Tacker M. (2021). Direct Comparison of the Lowest Effect Concentrations of Mutagenic Reference Substances in Two Ames Test Formats. Toxics.

[35] Matches J.R., Liston J. (1972). Effect of pH on low temperature growth of Salmonella. Milk Food Technol..

[36] Schreiner T., Ronzheimer A., Friz M., Morlock G.E. (2022). Multiplex planar bioassay with reduced diffusion on normal phase, identifying androgens, verified antiandrogens and synergists in botanicals via 12D hyphenation. Food Chem..

